# Guidelines for emergency laparoscopy

**DOI:** 10.1186/1749-7922-1-31

**Published:** 2006-10-19

**Authors:** Edmund AM Neugebauer, Stefan Sauerland

**Affiliations:** 1 Institute for Research in Operative Medicine, University of Witten/Herdecke, Cologne, Germany

## Abstract

Acute abdominal pain is a leading symptom in many surgical emergency patients. Laparoscopy allows for accurate diagnosis and immediate therapy of many intraabdominal pathologies. The guidelines of the EAES (European Association for Endoscopic Surgery) provides scientifically founded recommendations about the role of laparoscopy in the different situations. Generally, laparoscopy is well suited for the therapy of the majority of diseases that cause acute abdominal pain.

## Editorial

Emergency laparoscopy is widely used to identify the causative pathology of acute abdominal pain, often followed by laparoscopic treatment of the detected abdominal disorder. In the sequence of a series of a previous consensus development conferences, performed by our group under the mandate of the European Association for Endoscopic Surgery (EAES) since 1993 the most recent one (in 2005) aimed to develop guidelines to define, which subgroups of patients should undergo laparoscopic instead of open surgery for acute abdominal pain [1].

Emergency laparoscopy competes with the initial usage of other diagnostic procedures and imaging and additionally carries the risk of procedure-related complications especially in emergency situations, delay to define open surgical treatment and missing diagnosis. On the other hand laparoscopy offers a superior overview of the abdominal cavity with minimal trauma, always convertible to open surgery.

Clinical practice guidelines recommendations should be based on good scientific evidence from controlled clinical trials. The guidelines on laparoscopy for abdominal emergencies include the available evidence in this heterogeneous field. The responsible group of experts from different disciplines followed a transparent protocol with using a nominal group process for reaching consensus. They stated that all recommendations given are valid only for surgeons or surgical teams with sufficient expertise in laparoscopic surgery. Sufficient expertise however, is not defined although it is the most crucial factor to be taken into account.

Grade A recommendations (highest grade) for performing emergency laparoscopy and treatment are given for patients with a presumable diagnosis of perforated peptic ulcer, acute cholecystitis with the recommendation to carry out surgery as early as possible (< 48 hrs), acute appendicitis with treatment only if diagnosis is confirmed, but also in perforated cases and in a variety of suspected gynaecological disorders. Although highly recommended we should be aware of the fact, that in most of the studies in which hospital stay and convalescence were utilised as endpoints may merely reflect traditions of postoperative care and patient expectations associated with open procedures rather than differences between open and laparoscopic surgical techniques. Purists may argue that all studies need to be repeated using fast track rehabilitation programs in the open and laparoscopic arms.

The great number of diseases for which emergency laparoscopy achieved only a grade B or C recommendation, such as adhesive bowel obstructions, acute diverticulitis, non-biliary pancreatitis, hernia incarceration, suspected mesenteric ischemia or blunt and penetrating trauma calls for more clinical trials to be performed. For example in biliary pancreatitis there are no studies available comparing a wait-and-see policy versus early removal of bile duct stones, although the risk of a potentially life threatening recurrent pancreatitis when delaying bile duct clearance is generally considered to be unwarrantable. In complicated cases of acute diverticulitis (diffuse patients, stage III Hinchey classification) which usually requires emergency surgical exploration, only limited data are available for emergency laparoscopy. For incarcerated hernia laparoscopic surgery may be considered in emergency surgery cases. It seems, however, unjustified to adopt the principle of transferable evidence of the use of laparoscopy in inguinal, incisional or other hernias to the emergency situation. Further research is also needed in blunt abdominal trauma which prohibits a clear recommendation in favor of therapeutic laparoscopy for trauma. This list is by far not exhaustive but the few examples given should stimulate experienced academic laparoscopic surgeons to prove the evidence of their daily work.

In abdominal emergencies, abdominal pain is the leading symptom. Laparoscopic surgery aims to treat abdominal pain by the intervention. Most of the studies so far, however, did not measure acute pain and its relief with appropriate instruments, such as the visual – or the numerical rating scale, nor did they give adequate information on perioperative pharmalogical and non-pharmalogical pain treatment. It has been shown that adequate pain treatment is of significant value for the outcome of the patients in the acute phase but also to prevent chronic pain in the long run. These aspects need to be considered when performing future studies

In conclusion, the field of emergency laparoscopy, although difficult to perform, needs more and better performed controlled clinical trials. One may think that this is a usual conclusion of editorials like this, but this simply is the truth.
